# Low circulation of respiratory syncytial and influenza viruses during autumn-winter 2021 in the industrial workplace and long-term healthcare facilities in Athens, Greece

**DOI:** 10.3389/fmed.2022.1025147

**Published:** 2023-01-09

**Authors:** Eleni Papachristou, Chrysoula Rokka, Triantafyllia Sotiriadou, Leukothea Maneka, Alexandros Vassilakis, Spyros Sapounas, Dimitrios Paraskevis, Eddison Jahaj, Anastasia Kotanidou, Pagona Lagiou, Gkikas Magiorkinis

**Affiliations:** ^1^Department of Hygiene, Epidemiology and Medical Statistics, Medical School, National and Kapodistrian University of Athens, Athens, Greece; ^2^National Public Health Organization, Athens, Greece; ^3^First Department of Critical Care Medicine and Pulmonary Services, School of Medicine, Evangelismos Hospital, National and Kapodistrian University of Athens, Athens, Greece; ^4^Department of Epidemiology, Harvard T.H. Chan School of Public Health, Boston, MA, United States

**Keywords:** COVID-19, RSV, SARS-CoV-2, influenza, non-pharmaceutical interventions

## Abstract

The emergence of SARS-CoV-2 has pinpointed the importance of non-pharmaceutical interventions (NPIs), which have been traditionally used for the prevention of the spread of respiratory viruses among individuals. The aim of our study was to capture the level of circulation of respiratory syncytial and influenza viruses during a period of medium severity NPIs due to SARS-CoV-2 pandemics in Greece. A total of 2,225 nasopharyngeal samples were received during the year 2021 as a part of the routine diagnostic service and were divided into two study groups: (a) January to September 2021 and (b) October to the end of December 2021. The latter is the time of the year when there is a peak of infections from most respiratory viruses, and thus, most of the samples were tested in that period. The samples were taken from three different sites, i.e., (a) industrial workers in a factory, (b) elderly homecare facilities, and c) people who actively asked to be tested for SARS-CoV-2. All the samples were tested simultaneously for SARS-CoV2, RSV, and influenza virus. A total of 2,110 samples were negative for either of the three viruses, 106 were SARS-CoV-2-positive, and 9 were RSV-positive from which 7 were found in the workers’ group. None of the samples was found to be positive for the influenza virus, and no sample had co-infection. Our study shows the low-level circulation of RSV and influenza viruses during autumn-winter 2021 and will provide a reference for future studies of RSV and influenza in Greece.

## Introduction

There have been more than 70 years since the World Influenza Center in London was set up and started to collaborate with World Health Organization (WHO), in order to watch after the influenza outbreaks and answer the question of how epidemics arise and spread (1), and yet, this infection remains a major concern of public health worldwide. The great pandemic of the 19th century, which has been named “Asian Flu” (1889-1890) and was characterized by high morbidity and mortality rates, was followed by another pandemic, 30 years later, the so-called Spanish Flu. This pandemic was caused by the H1N1 virus, and the death toll of human lives (over 20 million) exceeded that of the victims of the First World War. Since then, many outbreaks of various intensities have occurred (2). Every year, more than half a million people die from the flu while about 5 million get infected (WHO). Influenza virus has a segmented single-stranded RNA genome virus with negative orientation, belongs to the Orthomyxoviridae family, and is divided into three types (A, B, and C). It causes acute respiratory disease that can occur repeatedly and affects people of all ages globally (3). While sporadic cases of the flu occur throughout the year, the epidemics break out generally in autumn-winter as the virus is more stable in lower temperatures, showing the greatest transmission rates at 5^0^C and the least above 30°C (4). The incubation period ranges from 1 to 4 days and transmission occurs by droplets from one infected person to another (5).

Another virus that causes acute respiratory infection in both children and adults is the respiratory syncytial virus (RSV), which affects almost 25 million people and leads to the death of almost 80,000 per year (6). RSV has been initially identified in 1956 during a study with throat materials from chimpanzees suffering from coryza (7, 8) and is a single-stranded negative sense RNA virus that belongs to the Paramyxovirus family and is classified in the Pneumovirus genus (9). Since then, this virus is frequently found in infants which has been well studied but is poorly defined in older patients (10) although they seem to be reinfected during their lifetime, which leads to high hospitalization and mortality rates (11, 12). Although in most cases, this reinfection is mild, immunosuppressed and frail elderly are at high risk of developing severe illness. The diagnosis of RSV infection in adults is more difficult than in young children in which it produces a well-characterized illness that is different from other wintertime circulating viruses (13).

The recent COVID-19 pandemic, which started as viral pneumonia in Wuhan, China and was caused by a new human-to-human transmissible coronavirus, the SARS-CoV-2 (14) posed an urgent need to apply non-pharmaceutical interventions (NPIs) such as frequent hand washing, continuous use of protective face masks, social distancing, avoidance of crowded places, and well-aired rooms in order to minimize the risk of being infected.

The “hottest” time of transmission for most respiratory tract viruses in the Northern Hemisphere begins around October as a result of the displacement of many activities indoors due to the temperature dropping. The period of October–December 2021 in Greece was characterized by less stringent NPIs compared to October–December 2020, when curfews and partial lockdowns were imposed. Relaxation of horizontal NPIs during October–December 2021 was feasible due to the implementation of the COVID-19 vaccination program; however, a mask mandate in closed spaces was still active throughout the study period. The aim of the study was to investigate whether NPIs that were in place for SARS-CoV-2 during October–December 2021 were likely to also mitigate the spread mode of other pathogenically important respiratory viruses, more specifically RSV and influenza. We note that there are no data available for previous RSV circulation in adults in Greece, and thus, our study offers valuable information about the level of circulation of these viruses during a period of NPI implementation and serves as a starting point for further research.

## Materials and methods

### Samples and study period

A total of 2,225 nasopharyngeal swab samples were tested as a part of a diagnostic preventive service for the SARS-CoV-2 pandemic in 2021. The analyzed tests were collected from three diagnostic routines, more specifically periodical routine screening of asymptomatic workers in an industrial setting, mitigation screening of asymptomatic elderly in nursing facilities where SARS-CoV-2 cases had been previously diagnosed, as well as people who actively looked for SARS-CoV-2 test. In all cases, the swabs came into the lab into a sterile transport tube containing the appropriate transport medium. A total of 1,390 (62.5%) samples were taken from industrial workers, 695 (31.2%) from homecare facilities, and the rest 140 (0.63%) were from people actively asking to be tested for SARS-CoV-2 from various sites of interest. Testing was intensified between October and December 2021 as we were expecting a higher incidence of RSV and influenza during this period, and thus, most of the samples (2,056/2,225, 92%) represent the fourth trimester of the year (1/10/21–31/12/2021). A Fisher’s exact test was performed to determine whether there was a significant change between sampling times. Here, we report a fully anonymized secondary analysis of the offered diagnostic service.

### RNA extraction and RT-PCR

For the isolation of the viral RNA, two commercially available RNA kits were used, namely, TANBead Nucleic Acid Extraction Kit (Taiwan Advanced Nanotech Inc., Taoyuan City, Taiwan) and QIASymphony DSP Virus/Pathogen Mini Kit (QIAGEN, Hilden, Germany), according to the manufacturer’s protocol.

The amplification was performed with the VIASURE SARS-CoV-2, flu, and RSV kit (CerTest Biotec, Zaragoza, Spain), which is a one-step real-time RT-PCR format, i.e., the reverse transcription and the amplification of the targeted area take place in the same tube. The target genes are as follows: for the SARS-CoV-2 virus, the N gene (N1 and N2), for the influenza virus (A/B), a conserved region of the M1 gene, and the RSV (A/B) virus, a conserved region of the N gene. The method uses gene-specific primers and fluorescent-labeled probes to distinguish the different amplicons.

## Results

Our analysis is composed of three epidemiologically interesting respiratory illness groups: first, a group of industrial workers, which represent a socially active group, likely to be living with their families (husband, wife, and children), second, a group of residents in homecare facilities, which represent the least active social group, but a high-risk respiratory transmission environment, and third, a group of persons actively seeking to be tested for SARS-CoV-2. In the first two groups, the testing was performed as a part of a preventive screening process on mostly asymptomatic persons.

In total, 2,110 (95%) of the total of 2,225 samples were negative for either of the three viruses. From the remaining 115 samples, 106 (92%) were SARS-CoV-2-positive and 9 (7.8%) were RSV-positive. None of the samples was positive for the influenza virus and no sample was found to have co-infection.

The majority of the SARS-CoV-2-positive samples were detected in homecare facilities where a positivity rate of 10% (Table 1) was observed for the period of interest. This is not surprising as a high SARS-CoV-2 positivity rate (6%) was also observed before October 2021 (Table 2), a finding compatible with the high transmissibility of SARS-CoV-2 within long-term healthcare facilities. Similarly, high rates of transmission were not observed for RSV even though there was clear evidence that the virus was circulating in the community (positive samples in industrial workers and actively seeking tests) and at least on one occasion, it was isolated from a patient in a homecare facility. Crucially, there was no other RSV-positive sample within the specific care home, even though the samples from other residents were also analyzed.

**TABLE 1:** Distribution of infections in each study group for the last trimester of the year 2021.

1/10/2021 – 31/12/2021	Total	SARS-CoV-2	RSV	Influenza
Industrial	1,374	1	7	0
Nursing home	614	65	1	0
Active test	68	3	1	0
Total	2,056	69	9	0

**TABLE 2:** Distribution of infections in each study group from January to September 2021.

1/1/2021 – 30/9/2021	Total	SARS-CoV-2	RSV	Influenza
Industrial	16	0	0	0
Nursing home	81	5	0	0
Active test	72	32	0	0
Total	169	37	0	0

In contrast, within the industrial screening samples, there was a constantly low prevalence of SARS-CoV-2 as a result of the active masking mandate within the working environment. Interestingly, 7 out of 9 RSV-positive samples (77.7%) were from industrial workers. From these, four were found positive in November and three in December 2021. Interestingly, two of the four positive samples from November were taken on consecutive days (8 and 9 November) while the other two samples were taken towards the end of November, 3 days apart the one from the other (21 and 25 November). The three positive samples from December are of interest too, as two of them were taken on the same day from the same working shift (2 December) while the other was taken the next day (3 December).

The people actively seeking to be tested for SARS-CoV-2 represent a diverse community group of people with a high risk for respiratory-related illness. Interestingly, in this group, we found one sample positive for RSV.

We performed Fisher’s exact tests to check for a significant change between sampling times. The tests were performed for RSV and SARS-CoV-2 separately and showed no significant differences for RSV. However, the lack of statistical significance for RSV is likely to reflect the lack of power due to the relatively low number of samples during the first testing period.

## Discussion

The current COVID-19 pandemic came not only as a new “headache” for mankind but also as a reminder of the effectiveness of traditional NPIs against respiratory tract viruses.

According to CDC,^1^ NPIs can be distinguished into three different kinds: personal, community, and environmental. Personal NPIs include frequent hand washing, covering coughs and sneezes, and staying at home in case of sickness. Community NPIs refer to social distancing in crowded places such as schools, workplaces, and places of worship and temporary closures when needed, while the environmental ones refer to routine surface cleaning of frequently touched surfaces from many people such as toys, refrigerators, and doorknobs.

Pharmaceutical interventions such as vaccination against SARS-CoV-2 have been a novelty in this pandemic as this virus was a new one. For RSV, there is yet no available and approved vaccine (15, 16) whereas there are more than 30 candidate vaccines as well as new monoclonal antibodies under evaluation (17). On the other hand, vaccination programs against the flu have been very beneficial both in preventing infection and in reducing complications and the need for hospitalization (18), but there have been still many cases every year.

In this study, nobody was found to be infected with the influenza virus although high circulation was expected as a result of the relaxed NPIs during the study period. Crucially, influenza virus infections were reported in Greece several months later, that is the first trimester of 2022 when there was a wider relaxation of NPIs (19). This fact is supported by the ECDC’s annual report which it is shown that the restrictions during the COVID-19 pandemic shortened the usual influenza season as it returned to the baseline levels earlier than in previous seasons (20).

On the other hand, we found nine persons to be infected with RSV. It is of interest that seven of them were workers in a large industrial site while only one was from elderly homecare facilities. This is compatible with RSV intra-family transmission where young children are usually the source of the infection. The seven positive samples in the industrial workers could be related to the transmission in the workplace as they were observed close to each other. On the other hand, social distancing restrictions in long-term healthcare facilities are likely to have contributed to the apparent lower circulation of RSV. This finding has important implications since RSV infection as the etiological agent of severe respiratory disease in adults has been underrated (21, 22). As it has been shown (23), the mortality and morbidity caused by RSV in older adults were comparable to or more severe than those due to influenza, resulting not only in longer hospitalization time (>7 days) but also in worse long-term survival for those infected by RSV than those with influenza.

The fact that we found one sample positive for RSV in the group of people who actively asked to be tested for SARS-CoV-2 suggests a potentially higher frequency for RSV in this group compared to the screening groups, although this could be a random finding due to the small size of this group. The lack of access to more data (demographic or any other kind) for this group restricts more information. These people asked to be tested either based on close contact with patients with SARS-CoV-2, a high-risk activity (e.g., unprotected social contact with many people), or due to having respiratory symptoms. Although there are many studies for RSV in infants and children in Greece that show a high incidence in this age group (24–26), the data for the adult population are limited so there is no baseline to compare these results to previous prevalence.

Although this study is limited by the size of the sample and the short observation period, it further supports that NPIs reduced the incidence of non-COVID-19 respiratory infections in general even during a period with a more relaxed NPIs setting. As the RSV period in Northern Hemisphere begins in November and expands to February of the oncoming year, the cases of RSV and flu infections during January and February 2022 could have been more than observed in this sample, which covered the calendar year until the end of December 2021. Our findings are further supported by studies (27–30) also showing the delay of the RSV re-appearance worldwide due to NPIs application. Given that NPIs have been gradually reversed, we expect that the incidence of respiratory infections in Autumn-Winter 2022 is likely to increase. Indeed, emerging evidence suggests that influenza viruses have been circulating during the summer of 2022 in Greece. Our study, thus, underpins the importance of maintaining compliance with NPIs in high-risk settings such as homecare facilities and hospitals.

The RSV virus has been found to circulate in the community and this could trigger the need for more well-organized studies in Greece, which not only will be useful from an epidemiological aspect by providing data that will fill the void that exists in the surveillance but also will serve as a basis for organizing the healthcare system to a point that will be able to support an outbreak.

## Data availability statement

The original contributions presented in this study are included in this article/supplementary material, further inquiries can be directed to the corresponding author.

## Ethics statement

The studies involving human participants were reviewed and approved by the Bioethics and Deontology Committee of the Medical School of the National and Kapodistrian University of Athens. Written informed consent for participation was not required for this study in accordance with the national legislation and the institutional requirements.

## Author contributions

GM designed the study and secured funding. EP wrote the first draft. EP, CR, TS, AV, and LM contributed to the experiments. SS, DP, EJ, AK, PL, and GM designed the sampling. All authors provided comments and approved the final version of the manuscript.

## Footnotes

1
https://www.cdc.gov/non-pharmaceutical-interventions/
